# First report of *Trichinella chanchalensis*, and detection of foreign *Trichinella spiralis,* in wildlife in Alaska

**DOI:** 10.1186/s13071-025-07142-x

**Published:** 2025-12-10

**Authors:** Cody J. Malone, Kimberlee Beckmen, Raphaela Stimmelmayr, Vladislav A. Lobanov, Maarten J. Voordouw, Jayne Ellis, Emily J. Jenkins

**Affiliations:** 1 Department of Veterinary Microbiology, Western College of Veterinary Medicine, University of Saskatchewan, 52 Campus Drive, Saskatoon, SK S7N 5B4 Canada; 2 Alaska Department of Fish and Game, Division of Wildlife Conservation, 1300 College Road, Fairbanks, AK 99701 USA; 3 Department of Wildlife Management, North Slope Borough, 1274 Agvik Street, Utqiagvik, AK 99723 USA; 4 Institute of Arctic Biology, University of Alaska Fairbanks, 2140 N Koyukuk Drive, Fairbanks, AK 99775 USA; 5 Centre for Food-Borne and Animal Parasitology, Canadian Food Inspection Agency, 116 Veterinary Road, Saskatoon, SK S7N 2R3 Canada; 6 Department of Veterinary Medicine, University of Alaska Fairbanks, 2141 Koyukuk Drive, 182 Arctic Health Research Building, Fairbanks, AK 99775 USA

**Keywords:** Alaska, Foodborne disease, Parasitology, *Trichinella*, Zoonotic disease

## Abstract

**Background:**

Members of the genus *Trichinella* are muscle-dwelling zoonotic parasites of global importance for public health, animal husbandry, and trade. *Trichinella chanchalensis* (T13) is the newest species in the genus, first described in the Yukon and the Northwest Territories, for which the geographical distribution remains unknown due to limitations of the gold standard test for genotyping (multiplex polymerase chain reaction [PCR]). Our primary objective was to determine whether *T. chanchalensis* was present in Alaska, using a new molecular method that enables the description of the prevalence, co-infection, host associations, and risk factors for *Trichinella* spp. infection in wild carnivores.

**Methods:**

*Trichinella* spp. larvae were recovered through artificial digestion of muscle and genotyped using next-generation sequencing (NGS).

**Results:**

*Trichinella* spp. larvae were detected in 53/157 (34%) animals, namely wolverines (*Gulo gulo*), arctic foxes (*Vulpes lagopus*), red foxes (*Vulpes vulpes*), coyotes (*Canis latrans*), wolves (*Canis lupus*), brown bears (*Ursus arctos*), and polar bears (*Ursus maritimus*), but not in black bears (*Ursus americanus*) or lynx (*Lynx canadensis*). Prevalence was highest in polar bears and wolverines, while intensity (larvae per gram, LPG) was highest in red foxes, arctic foxes, and wolves. Most animals (65%) harbored single infections with *Trichinella nativa*, followed by mixed infections of *T. nativa* and *Trichinella* T6 (33%). A single wolverine was infected with *T. nativa*, T6, and *T. chanchalensis*. Combining NGS with statistical methods, we found no evidence of competition between *T. nativa* and T6 in host muscles. *Trichinella* spp. infection (primarily *T. nativa*) was the highest in the Northwestern region, whereas T6 infection probability was higher in the Interior and Southern regions, suggesting differences in environmental resistance even among these three taxa. In a single, highly infected brown bear, we detected a rare case of *Trichinella spiralis* of foreign origin based on whole-genome sequencing, suggesting illegal importation and disposal of meat.

**Conclusions:**

We report a new geographical record for *T. chanchalensis* and a rare finding of *T. spiralis* in North American wildlife, and demonstrate the utility of new NGS methods for describing the ecology of parasites maintained in wildlife hosts commonly presenting as co-infections.

**Graphical Abstract:**

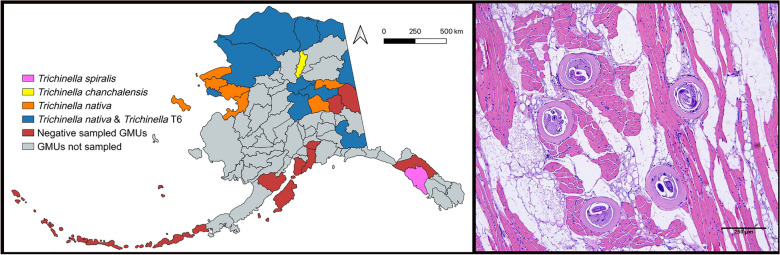

## Background

*Trichinella* is a genus of muscle-dwelling parasitic nematodes of zoonotic importance, which do not have a true environmental or free-living stage, making transmission entirely dependent on carnivory or scavenging [1]. Globally, there are 13 recognized *Trichinella* taxa. To date, three have been detected in Alaskan wildlife: *Trichinella spiralis* (T1), *Trichinella nativa* (T2), and *Trichinella* T6 [2–6]. *Trichinella nativa* and T6 are common in arctic wildlife in North America, and are freeze-tolerant, which allows viable larvae to persist longer in carcasses and eliminates freezing as a food safety intervention [1, 7, 8]. A recent outbreak in three US states linked to frozen black bear (*Ursus americanus*) meat harvested in Canada emphasizes that trichinellosis remains a public health concern [9]. The newest species, *Trichinella chanchalensis*, has been found in carnivores including wolverines (*Gulo gulo*), American marten (*Martes americana*), wolves (*Canis lupus*), lynx (*Lynx canadensis*), and coyotes (*Canis latrans*) from the Northwest Territories and the Yukon, Canada [10–12].

Given that the Yukon and Alaska share a border, our main objective was to determine whether *T. chanchalensis* is present in Alaska, and to assess the zoonotic risk of *Trichinella* spp. transmission to humans. Humans become infected with *Trichinella* spp. when consuming meat from carnivores (such as wildlife and swine) raw or prepared using methods unreliable for inactivating the larvae. The risk of human infection in Alaska (4.1 cases per million) is 40 times that in the rest of the United States (0.1 cases per million), likely from higher consumption of wildlife [13]. Therefore, we used next-generation sequencing (NGS) to describe the abundance, diversity, and biogeography of *Trichinella* spp. in Alaskan wildlife.

## Methods

A total of 157 legally harvested, found dead, or euthanized wild carnivores collected during 2008–2024 from 27 of 73 game management units (GMUs) were voluntarily submitted to the wildlife health programs of the Alaska Department of Fish and Game (ADFG) and North Slope Borough Department of Wildlife Management (NSB DWM). Once carcasses were received by ADFG or NSB DWM, they were kept frozen until necropsy, and muscle samples collected during necropsy were re-frozen at −20 °C until processing. Age and sex were determined during necropsy, and harvest location was submitted by the hunter/harvester/conservation officer. Animals were legally harvested for purposes other than research and were therefore exempt from research ethics approval, and permits were obtained to ship wildlife samples across borders (University of Saskatchewan Exemption #010, Canadian Food Inspection Agency [CFIA] import permit #A-2023-00669-1, Convention on International Trade in Endangered Species of Wild Fauna and Flora [CITES] permit 24US64147R/9).

A single muscle was analyzed for 97 animals (76 tongue, 15 diaphragm, five masseter, one temporalis), and multiple muscles were combined for 60 animals (11 tongue and diaphragm, 49 tongue, diaphragm, and masseter) for a median weight of 10.7 g per animal (0.83–27.11 g). Larvae were recovered and quantified using the artificial digestion method with HCl and pepsin [14]. Up to 100 (range 3–100) larvae per animal were stored in GeneAmp 1× polymerase chain reaction (PCR) buffer (Thermo Fisher Scientific, Carlsbad, CA, USA) at −80 °C until DNA extraction.

DNA was extracted from pools of *Trichinella* spp. larvae from 53 individual animals and six negative DNA extraction controls (nuclease-free water) using the PureLink Genomic DNA Mini Kit (Thermo Fisher Scientific, Carlsbad, CA, USA) following the manufacturer’s instructions. Pools of larvae from each animal were genotyped using amplicon sequencing targeting the internal transcribed spacer 1 (ITS-1) region of the ribosomal RNA cistron, which allows for differentiation of all North American taxa [10].

After quantifying using a Qubit High-sensitivity double-stranded DNA (dsDNA) kit (Thermo Fisher Scientific), pooled libraries were diluted to 8 pM, mixed 3:1 with PhiX control v3 (Illumina) and sequenced using MiSeq 500-cycle v2 nano kits on the Illumina MiSeq platform (Illumina, San Diego, CA, USA). Sequences were analyzed using a customized Dada2 pipeline and three different classifiers (IDTaxa, AssignTaxonomy, and BLASTn) in RStudio (v. 2023.09.1+494) [15–17].

From a brown bear (*Ursus arctos sitkensis*) infected solely with *T. spiralis*, DNA was extracted from pooled larvae as above, except the Proteinase K digestion step was extended overnight. Whole genomes were sequenced using Illumina DNA Prep^®^ kits and an Illumina MiSeq. Paired-end reads were trimmed for quality, mapped to published reference sequences (mitochondrial DNA [mtDNA]: NC_002681 and ribosomal DNA [rrDNA]: MW302168), and consensus sequences were generated using previously published parameters [18]. Comparisons with published sequences were accomplished by phylogenetic analysis of neighbor-joining trees with 100 bootstrap replicates built from aligned sequences using the Jukes–Cantor substitution model.

### Statistical analysis

The EpiTools epidemiological calculator [19] was used to calculate *Trichinella* spp. infection prevalence (proportion of animals infected with any *Trichinella* taxa) with 95% confidence intervals (CI) for each host group, age, sex, and location (GMU). Infection intensity was calculated as larvae per gram (LPG) of muscle digested and reported as median (range) for each animal species, not including negative animals. Abundance was calculated by multiplying relative proportions of each *Trichinella* taxon on NGS by LPG as reported previously [12], and included negative animals.

*Trichinella* spp. infection status (0 = negative, 1 = positive) was initially analyzed using generalized linear models (GLMs) with binomial errors, but due to some host groups having very low or very high infection prevalence, the GLMs were unable to estimate the parameters. Therefore, *Trichinella* spp. infection status was analyzed using Firth’s penalized logistic regression. Fixed factors were *Trichinella* taxa (*T. nativa* and T6), host group (wolverines; lynx; ursids—black, brown, and polar bears; vulpines—arctic and red foxes; and other canids—wolves and coyotes), age group (juveniles, subadults, and adults), sex, and location groups based on ADFG GMUs. The 73 GMUs were grouped into three regions (Northwestern, Interior, and Southern). To determine whether there were interactions between *T. nativa* and T6, we included the presence of T6 as an explanatory factor for *T. nativa* infection status, and vice versa.

Parasite abundance was analyzed using GLMs with Poisson or negative binomial errors. For the GLMs with Poisson errors, model residuals were highly over-dispersed, whereas the GLMs with negative binomial errors failed to converge. For this reason, parasite abundance was log_10_(X + 1)-transformed and analyzed using linear models (LM) with normal errors.

## Results

**Fig. 1:**
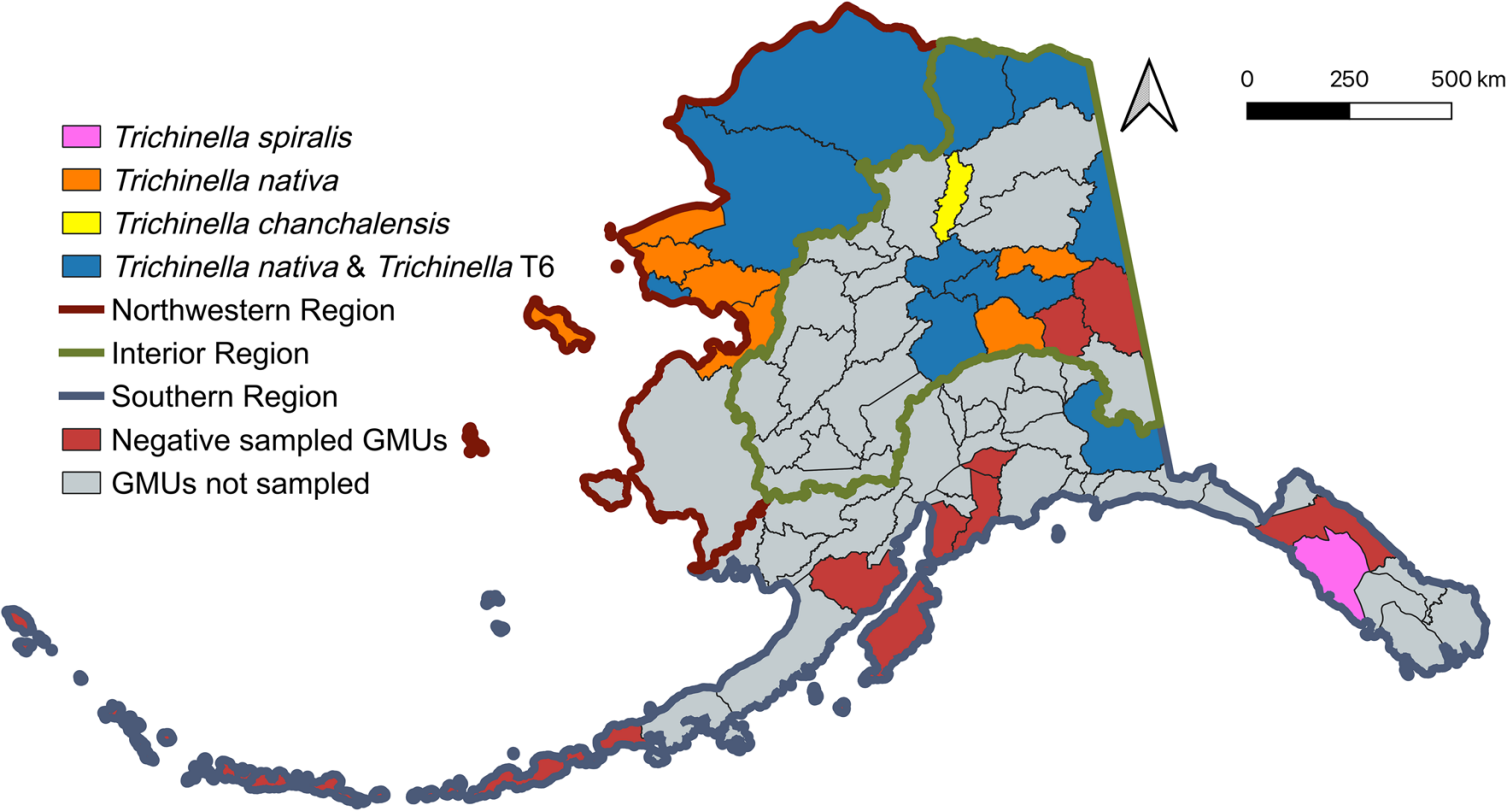
Detections of larvae of *Trichinella* species/genotypes in muscles of wildlife in game management units (GMUs) of Alaska. Map created with QGIS (v3.42.1-Münster)

*Trichinella* spp. larvae were detected in 34% (53/157) of hosts examined from nine species of wildlife; larvae were not detected in black bears or lynx (Table 1). Of host species with *n* > 10, prevalence was highest in wolverines, brown bears (*U. arctos*), and red foxes (*Vulpes vulpes*), while median infection intensity was highest in arctic foxes (*Vulpes lagopus*), red foxes, and wolves. Of the sampled GMUs, 67% (18/27) had at least one animal positive for larvae of *Trichinella spp*. (Fig. 1).

**Table 1:** Prevalence, intensity, and diversity of *Trichinella* spp. in muscles of wild carnivores from Alaska (*n* = 157), organized in descending order of sample size

Host species	No. positive/no. sampled (percent positive, 95% CI)	Median infection intensity (range) in larvae per gram	*Trichinella* taxa proportions
Red fox (*Vulpes vulpes*)	17^a^/39 (44%, CI 29–59%)	29.1 (0.4–206.5)	T2: 13/16 T2/T6: 3/16
Wolverine (*Gulo gulo*)	17/32 (53%, CI 37–69%)	3.9 (0.3–373.9)	T2: 7/17 T2/T6: 9/17 T2/T6/T13: 1/17
Grey Wolf (*Canis lupus*)	7/28 (25%, CI 12–44%)	8.4 (2.0–18.6)	T2: 5/7 T2/T6: 2/7
Arctic fox (*Vulpes lagopus*)	2/17 (12%, CI 2–36%)	51.6 (27.7–75.6)	T2: 1/2 T2/T6: 1/2
Black bear (*Ursus americanus*)	0/13 (0%, CI 0–27%)	0	ND
Brown bear (*Ursus arctos*)	6^a^/12 (50%, CI 25–75%)	6.5 (0.2–379.9)	T1: 1/5 T2: 2/5 T2/T6: 2/5
Lynx (*Lynx canadensis*)	0/10 (0%, CI 0–32%)	0	ND
Polar bear (*Ursus maritimus*)	3/3 (100%, CI 38–100%)	7.8 (0.5–26.7)	T2: 3/3
Coyote (*Canis latrans*)	1/3 (33%, CI 6–80%)	5.9	T2: 1/1

*T1* *T. spiralis*, *T2* *T. nativa*, *T6* *Trichinella* T6, *T13* *T. chanchalensis*, *ND* no data

^a^One red fox and one brown bear larval pool failed to amplify

*Trichinella* spp. larvae from 96% (51/53; two failed to amplify) of the positive animals were successfully genotyped as *T. nativa*, T6, *T. spiralis,* or *T. chanchalensis*. The infection prevalence of *T. nativa* (30%) was ~ 3 × higher than that of T6 (11%). Single infection with *T. nativa* (63%; 32/51) was the most common, followed by *T. nativa*/T6 co-infection (33%; 17/51). *Trichinella nativa*/T6/*T. chanchalensis* co-infection was detected in one wolverine from north central Alaska (GMU 24A), and *T. spiralis* as a single infection in one brown bear from southeastern Alaska (GMU 4) (Fig. 1).

Of 890,057 merged, quality-filtered reads from all samples across three sequencing runs, 865,341 reads were assigned to a *Trichinella* taxon: 708,212 as *T. nativa*, 130,326 as T6, 13,612 as *T. spiralis*, and 13,191 as *T. chanchalensis*. The median number of reads per sample was 15,975 (4,758–25,602). All negative extraction controls had ≤ 1 read assigned to *Trichinella* taxa.

### *Trichinella* spp. infection status and abundance

Animals from the Northwestern region had the highest predicted *Trichinella* spp. infection probability (43%, 95% CI: 18–71%), followed by the Interior (25%, 95% CI: 12–44%) and Southern regions (8%, 95% CI: 2–31%). However, none of the pairwise differences between regions were significant (all *P* > 0.06). Adults had a significantly higher infection probability (39%, 95% CI: 22–59%) than juveniles (14%, 95% CI: 4–35%; odds ratio [OR] = 0.25 ± 0.13, *P* = 0.023). Larval abundance (mean ± standard error [SE]; units of *Trichinella* spp. LPG) was significantly influenced by location (*P* = 0.0007) and weight of muscle (*P* = 0.016). Animals from the Northwestern region had the highest *Trichinella* spp. abundance (12.490 ± 4.790), followed by the Interior (2.477 ± 0.918) and Southern regions (0.592 ± 0.747). *Trichinella* spp. abundance was significantly higher in the Northwestern region than the Southern region (*P* = 0.04) (Table 2).

**Table 2:** Descriptive statistics for risk factors for *Trichinella* infection and abundance in Alaskan carnivores, with significantly higher prevalence in adults in the Northwestern region

Risk factors	*Trichinella* taxa prevalence and 95% CI
Age	
Juveniles	7/42 (17%, CI 8–31%)
Subadults	5/27 (19%, CI 8–37%)
Adults	35/77 (45%, CI 35–57%)
Sex	
Male	25/70 (36%, CI 26–47%)
Female	22/76 (29%, CI 20–40%)
Harvest location	
Northwestern	24/43 (56%, CI 41–70%)
Interior	20/78 (26%, CI 17–36%)
Southern	3/25 (12%, CI 3.3–31%)

### *Trichinella nativa* infection status and abundance

*Trichinella nativa* infection probability was highest in the Northwestern region (92%, 95% CI: 70–98%), followed by the Interior (73%, 36–93%) and Southern regions (21%, 2–75%). Odds of *T. nativa* infection were significantly higher in the Northwestern region than in the Interior (OR = 4.36 ± 1.99, *P* = 0.004) and Southern regions (OR = 44.01 ± 58.8, *P* = 0.015). The abundance of *T. nativa* (mean ± SE; units of *T. nativa* LPG) was highest in the Northwestern region (4.122 ± 1.230), followed by the Interior (1.224 ± 0.452) and Southern regions (0.491 ± 0.466). Pairwise differences showed significantly higher abundance in the Northwestern region than both the Interior (*P* = 0.034) and Southern regions (*P* = 0.007).

### *Trichinella* T6 infection status and abundance

T6 infection probability was highest in the Southern region (46%, 95% CI: 10–87%), followed by the Interior (7.4%, 2–24%) and Northwestern regions (1.7%, 0.3–10%). The pairwise differences between the Northwestern and Southern regions approached significance (OR = 0.0198 ± 0.0328, *P* = 0.051). T6 abundance (mean ± SE; units of T6 LPG) was highest in the Interior region (0.330 ± 0.071), followed by the Southern (0.288 ± 0.123) and Northwestern regions (0.099 ± 0.070). The difference between the Northwestern and Interior regions approached significance (*P* = 0.07).

### Interactions between *T. nativa* and T6: infection status and abundance

The probability of *T. nativa* infection was significantly higher in T6-positive animals (97%, 95% CI: 65–100%) than in T6-negative animals (13%, 6–27%; *P* = 0.0007). Conversely, the probability of T6 infection was higher in *T. nativa*-positive animals (51%, 95% CI: 25–77%) than in *T. nativa*-negative animals (0.5%, 95% CI: 0.1–6%, *P* = 0.0006). The abundance of *T. nativa* was almost significantly higher in T6-positive individuals (estimated marginal mean [EMM] = 2.602 ± 1.230 *T. nativa* LPG) than T6-negative individuals (0.834 ± 0.233 *T. nativa* LPG; *P* = 0.059). Conversely, T6 abundance was significantly higher in *T. nativa*-positive individuals (EMM ± SE = 0.570 ± 0.115 T6 LPG) than in *T. nativa*-negative individuals (–0.029 ± 0.0453 T6 LPG; *P* < 0.0001).

### *Trichinella spiralis*-infected brown bear

**Fig. 2:**
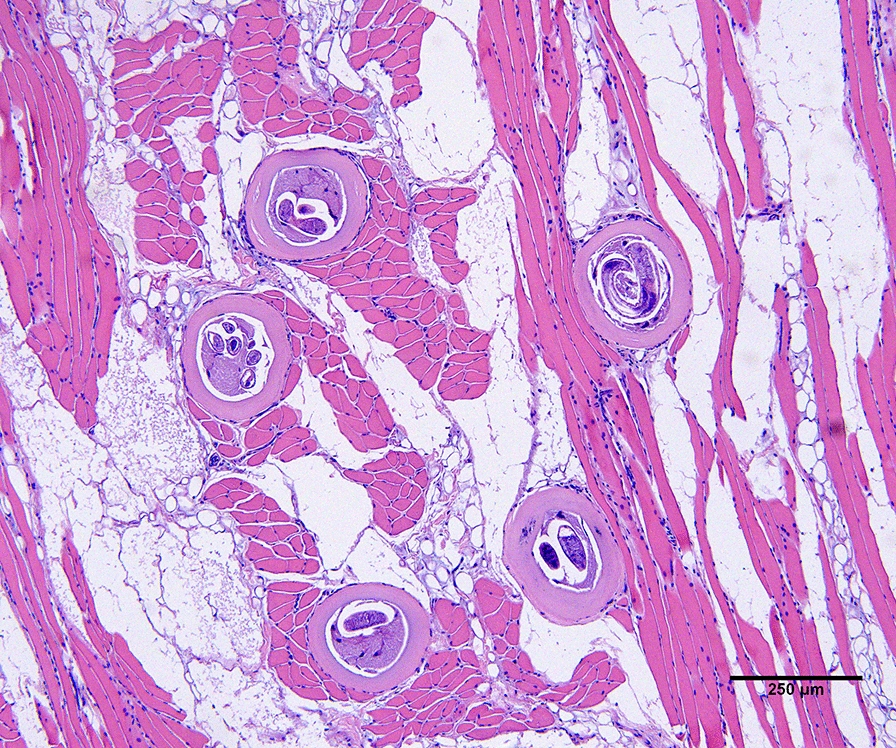
The histological image shows the infective encysted larval stage of *Trichinella spiralis* in the tongue of a heavily infected Alaskan brown bear. Coiled larvae are seen in both longitudinal and cross sections within host muscle cells (nurse cells)

**Fig. 3:**
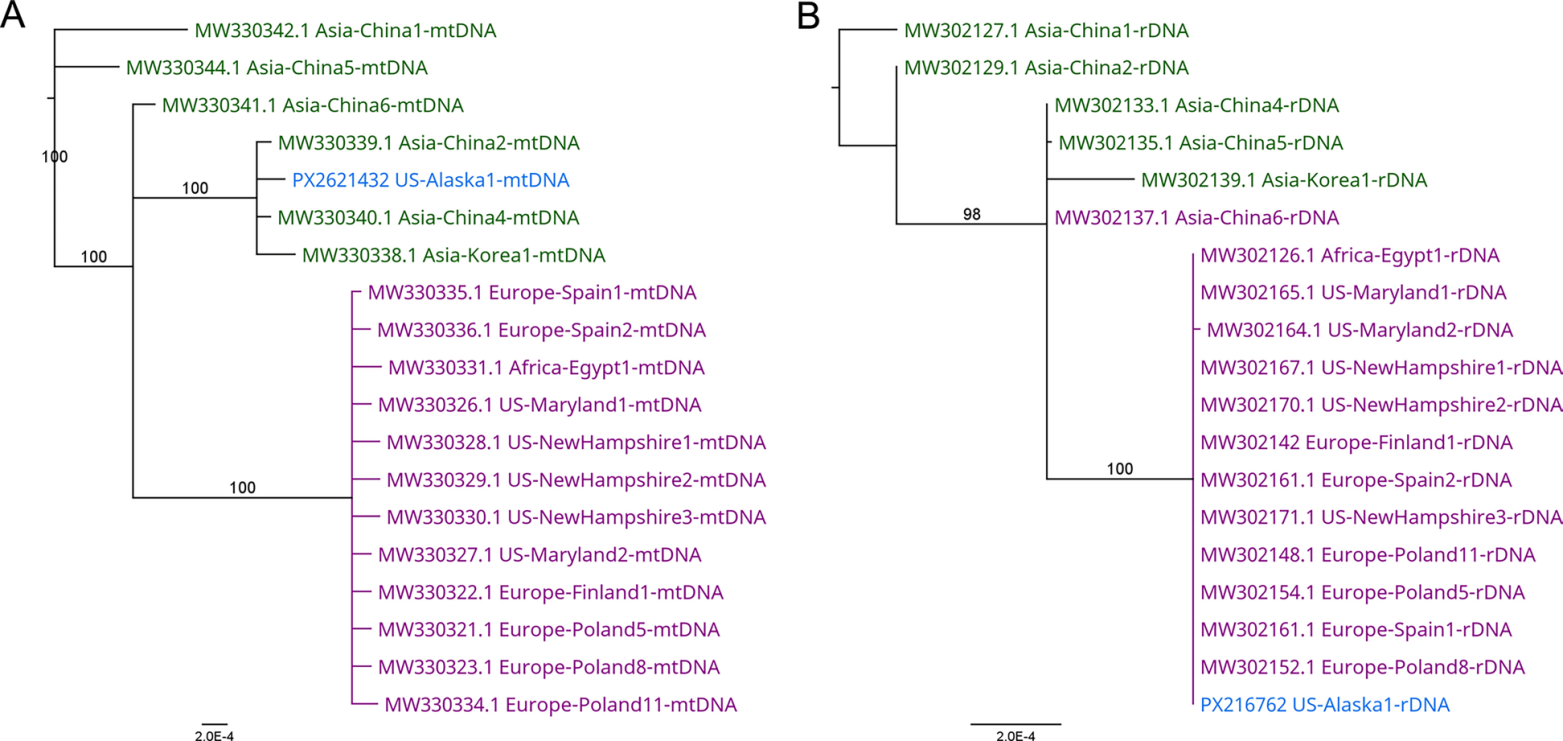
Phylogenetic trees of *T. spiralis* isolated from an Alaskan brown bear. **A** Mitochondrial DNA phylogenetic tree, **B** Ribosomal DNA phylogenetic tree. Color coding: green = Asian *T. spiralis* isolates; purple = European and North American *T. spiralis* isolates; blue = Alaskan bear *T. spiralis* isolate from this study

One 3-year-old male brown bear was infected with 380 LPG of *T. spiralis* (Fig. 2). Over 50% of larvae recovered from this bear were tightly coiled (an indicator of larval viability), despite being frozen in muscle for 3.5 years. No motility was observed in four aliquots of 25 larvae in phosphate-buffered saline (PBS) incubated at 37 °C for 30 min. Whole-genome sequencing generated 4,371,934 paired-end reads; after trimming for high quality, 1,430,514 reads remained. Of these, 35,888 and 2,007 reads mapped to the mitochondrial genome (mtDNA) and rDNA. More than 99% of consensus base calls had cumulative quality scores greater than Q40. Ribosomal DNA was 99.8% similar to isolates from the USA and Western Europe, with an average of 19 single-nucleotide polymorphisms (SNPs) across 7726 base pairs, and was 99.52% similar to isolates from Asia, with 39 SNPs. In phylogenetic analysis using rDNA, the Alaskan isolate from the brown bear grouped with western isolates with 100% bootstrap support (Fig. 3A). In contrast, mtDNA was 99.98% similar to isolates from Asia, with only seven SNPs from three Asian isolates, compared with 99.65% similarity and 48 SNPs with western isolates. In the neighbor-joining tree analysis using mtDNA, the Alaskan *T. spiralis* clustered with Asian isolates with 100% bootstrap support (Fig. 3B).

## Discussion

We detected *T. chanchalensis* for the first time outside of Canada, representing a new geographical record. In wild carnivores from Alaska, single infections with *T. nativa* were the most common, followed by *T. nativa*/T6 co-infections, which were much less common than in Yukon carnivores [12]. We identified three taxa endemic to northern North America (*T. nativa*, T6, and *T. chanchalensis*), each remaining genetically distinct despite sympatry and shared hosts, suggesting limited interspecific gene flow and stable lineage separation within northern ecosystems. We also discovered a rare single infection with *T. spiralis* in a brown bear from a rural island in Alaska, which is unusual, given that this parasite is typically associated with swine and is thought to be eliminated in the USA and Canada [20, 21]. In addition, the genotype of this isolate implicated foreign origin, possibly through long-distance anthropogenic dispersal. Our NGS approach enabled better detection of underrepresented *Trichinella* taxa and allowed for competition and abundance analyses not previously possible with traditional methods. However, storage time in the freezer (up to 16 years) and freeze–thaw cycles may have decreased larval quality, especially for species that do not survive freezing. Isolates from more recent infections or of taxa with better-preserved genetic material may have been preferentially amplified.

Our study prevalence (34%; 53/157) of *Trichinella* spp*.* in Alaskan carnivores was higher than 11.7% of carnivores previously reported in a much larger study of 2433 Alaskan carnivores (brown, black, and polar bears; arctic and red foxes; wolves; coyotes; wolverines; and lynx) [22], but lower than the 74% observed in a recent study using the same methods in the Yukon [12]. Host-specific prevalence in our study was variable, and some species had small sample sizes, but prevalence was highest in wolverines (53.1%) and brown bears (50%). The parasite was not detected in lynx despite detections in 21% of Alaskan lynx previously [6]. Prevalence was the lowest for black bears (0%), intermediate for brown bears (50%), and highest for polar bears (100%), reflecting increasing levels of carnivory. In canids, our finding of 25% (7/28) in wolves was slightly lower than the 36% prevalence in 148 wolves from the interior of Alaska [23]. Our prevalence in arctic foxes at 12% (2/17) was similar to the 7.2% found by Rausch et al. [22] and ~16% by Seymour et al. [5].

Arctic and red foxes had the highest median infection intensity of 51.6 and 29.1 LPG in the current study, which may reflect consumption of offal and meat scraps from harvested wildlife discarded in landfills. On Alaska’s North Slope, the arctic fox diet is primarily marine-based; however, near Prudhoe Bay—where arctic foxes from the current study were harvested—their diet involves more anthropogenic foods [24, 25]. Similarly, red fox diets are largely dominated by species of low importance for *Trichinella* spp. transmission (lemmings and voles), but anthropogenic food consumption by red foxes near communities has also been reported [25]. Median intensity in brown and polar bears was less than 10 LPG, but even one LPG is considered a food safety risk [26].

We report the first geographical record for *T. chanchalensis* outside of Canada in a single wolverine from north central Alaska. The prevalence of *T. chanchalensis* (0.6%; 1/157) was considerably lower than the 3% and 19% reported from wolverines and other species in the Yukon [12, 27], which was unexpected, as Alaska and Yukon share a 752-mile border [28] and ecoregions with similar physiographic, climatic, and biological characteristics [29]. These findings contradict a hypothesis that the presence of *T. chanchalensis* in North America is a result of a Beringian dispersal from Eurasia, based on observation of a westward prevalence gradient in northwestern Canada [27]. Much of the Alaskan interior and western-interior Yukon are plateaus with rolling hills, which would not be a significant barrier to animal movement [30, 31]. Although some geographical barriers exist, such as the Wrangell and St. Elias Mountain ranges in southeastern Alaska/southwestern Yukon, animals such as the Porcupine caribou herd and associated wolves and wolverines (both known hosts for *T. chanchalensis*) move between Alaska and the Yukon [12, 32–34]. Since host species, and therefore *Trichinella* spp., are not completely isolated between Alaska and the Yukon, there must be other barriers limiting the establishment of *T. chanchalensis* in Alaska.

A brown bear heavily infected with *T. spiralis* was found on Chichagof Island, Alaska, near Freshwater Bay. This was an unexpected finding for several reasons. While *T. spiralis* has been reported previously in a brown bear from an unspecified location in Alaska [2], this parasite is rare in wildlife, primarily associated with swine, and considered eliminated from commercial swine production in the USA and Canada [21]. Domestic pigs are also not commonly kept in Alaska, and there are no backyard pig farms located on Chichagof Island. The nearest communities, Tenakee Springs and Hoonah, are approximately 10 miles away and are not known for swine-rearing; therefore, this bear was likely infected by consuming meat or animals imported from elsewhere, such as food waste from a cruise ship discarded in a community landfill.

This is further reinforced by the characterization of rDNA and mtDNA, which suggest an American and Asian origin, respectively. This isolate could be a hybrid between an American/European *T. spiralis* male and an Asian *T. spiralis* female; however, we would have expected higher levels of heterozygosity in the rDNA. Therefore, this isolate is likely a recent arrival to North America with an Asian origin. *Trichinella* spp. have been under-sampled in Asia, and consequently, there is likely more genetic diversity than currently described, which could explain the observed genotype. This finding argues for more systematic sampling of *Trichinella* spp. in Asia to better understand genetic diversity, elsewhere to determine whether the Asian mtDNA haplotype is no longer confined to Asia, and locally in southeastern Alaska to determine whether *T. spiralis* is present in other wildlife.

Adult animals had a significantly higher prevalence of *Trichinella* spp. than juvenile animals, which is expected with more feeding opportunities to become exposed. Probability of infection and larval abundance of *Trichinella* spp. infection generally, and *T. nativa* specifically, were highest in the Northwestern region, with trends suggesting a spatial gradient (north to south). This suggests that *T. nativa* may be better adapted to the more extreme Arctic coastal tundra climate [35, 36]. Alternatively, this may reflect a higher proportion of marine mammals in the diet of carnivores in the Northwestern region, as *T. nativa* is the only species known to infect marine mammals in northern North America [37]; however, the role of marine mammals in transmission is likely very low [5]. It is also possible that other ecological factors, such as host density or scavenging behaviors, drive regional differences in *T. nativa* prevalence and abundance.

Across Alaska, *T. nativa* had higher infection probability and abundance than T6. This pattern was reversed in the Yukon, where T6 was markedly more abundant [12]. Within Alaska, T6 was most prevalent and abundant in the Southern and Interior regions, indicating more favorable environmental conditions and host communities. Unlike *T. nativa*, we found some evidence of host specificity for T6, as the prevalence was significantly higher in wolverines than in other carnivores. In contrast, our previous study in the Yukon found no evidence that either *T. nativa* or T6 was specialized in different host species [12]. The geographical distribution of *T. chanchalensis* in Alaska, Yukon, and the Northwest Territories appears to more closely resemble the distribution of T6, with a preference for cold climates, but not Arctic tundra, where *T. nativa* often dominates [11, 12].

The presence of T6 had a significant positive effect on the abundance of *T. nativa*, and conversely, the presence of *T. nativa* had a positive, although not significant, effect on the abundance of T6. Therefore, these data do not support the hypothesis of competitive exclusion between *T. nativa* and T6, which agrees with our findings from the Yukon [12]. A simple explanation for the positive association between the two *Trichinella* taxa is that mechanisms favorable for the transmission of *T. nativa* are also favorable for T6.

From a public health perspective, one-third of terrestrial carnivores tested in Alaska were infected with *Trichinella* spp., which means that the parasite is circulating in sylvatic reservoirs. Black bear meat has been implicated in many human outbreaks in North America [9, 38–44], where it continues to be the most common source of human infection [37]. It is reassuring that we did not detect *Trichinella* spp. in black bears or in lynx—which are also consumed in some areas [45]. Other host species in this study (such as red fox) are less commonly consumed and pose less of a threat despite higher prevalence and intensity. Nevertheless, consumption of carnivore meat from adult animals in the Northwestern region poses the highest public health risk, based on trends in prevalence and abundance. All three polar bears that we tested were infected, as was a single polar bear in another study from Alaska [5], and this species should be considered a very high risk for foodborne transmission unless cooked thoroughly, as is practice in many local communities. The detection of *T. spiralis* in the brown bear is also concerning, as *T. spiralis* is historically of high public health and trade significance.

## Conclusions

We conducted an Alaska-wide surveillance study on *Trichinella* spp. in wildlife, contributing new data to the geographical and host range of these zoonotic parasites, including three endemic northern taxa (*T. nativa*, T6, and *T. chanchalensis*), as well as *T. spiralis*. Future studies should focus on determining whether introduced genotypes of *T. spiralis* are present in other wildlife in southeastern Alaska and on expanded testing of wildlife in areas not sampled in the current study to enhance our understanding of *Trichinella* spp. distribution, transmission, and diversity. *Trichinella* spp. transmission is shaped by host ecology, regional climate, and anthropogenic factors, with distinct species showing geographical and, in the case of T6, potential host-specific patterns that warrant further research effort.

## Data Availability

Datasets generated or analyzed during this study are available from the corresponding author on reasonable request. Sequence data for mitochondrial and ribosomal loci can be found in GenBank as accession numbers PX2621432 and PX216762, respectively. Raw sequencing data is stored as SRA files in SRR35097920, SRR35097919, and SRR35097921 in BioProject: PRJNA1309764 and BioSample: SAMN50752979.
